# Supplementary material for: Quantitative Monitoring of Cyclic Glycine–Proline in Marine Mangrove-Derived Fungal Metabolites
Source: J Fungi (Basel). 2024 Nov 10;10(11):779. doi: 10.3390/jof10110779 (PMC11595644; doi:10.3390/jof10110779)
Supplement: Supplementary file 1 [file jof-10-00779-s001.zip › jof-3294947-supplementary.pdf]

SUPPLEMENTARY MATERIAL

Quantitative Monitoring of Cyclic Glycine-Proline in Marine Mangrove-Derived Fungal Metabolites

Contents

Figure S1. Full scan mass spectrum of cGP standard
Figure S2. Product scan mass spectrum of cGP standard
Table S1. SRM parameter optimization results
Figure S3. SRM chromatogram of the standard at a concentration of 0.14 μg/mL
Figure S4. SRM chromatogram of the blank control
Table S2. Screening of wild-type fungal strains from marine mangroves
Figure S5. SRM chromatogram of the *Penicillium pedernalense*
Figure S6. SRM chromatogram of the *Penicillium steckii*
Table S3. The limits of detection (LOD) and the limits of quantitation (LOQ)
Table S4. Calibration curve of standard
Table S5. The result of Precision
Table S6 The result of Repeatability
Table S7. The result of Recovery

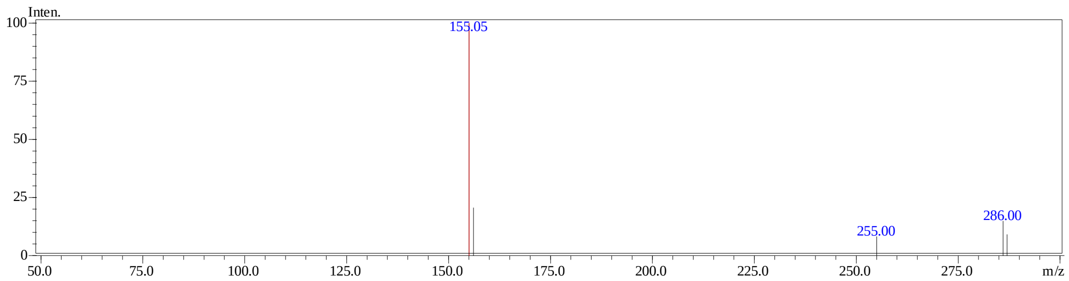

Figure S1. Full scan mass spectrum of cGP standard

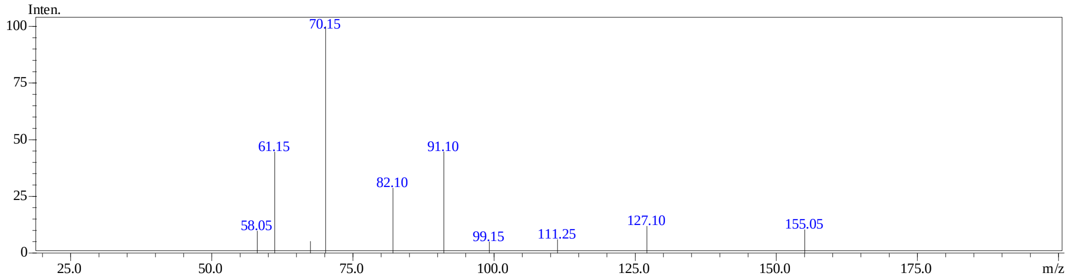

Figure S2. Product scan mass spectrum of cGP standard

Table S1. SRM parameter optimization results

Analyte	Precursor ion (*m/z*)	Detection mode	SRM (*m/z*)	Dwell time (msec)	Q1 Pre deviation (V)	Collision energy (eV)	Q3 Pre deviation (V)
cGP	155.00	+	70.10	100.00	-12.0	-22.0	-13.0
			82.10	100.00	-12.0	-21.0	-15.0

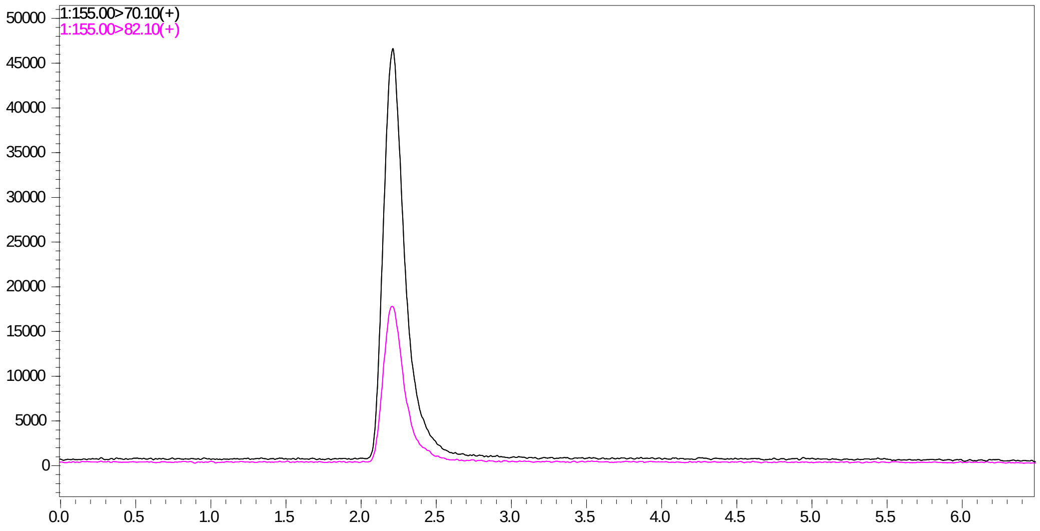

Figure S3. SRM chromatogram of the cGP standard at a concentration of 0.14 μg/mL.

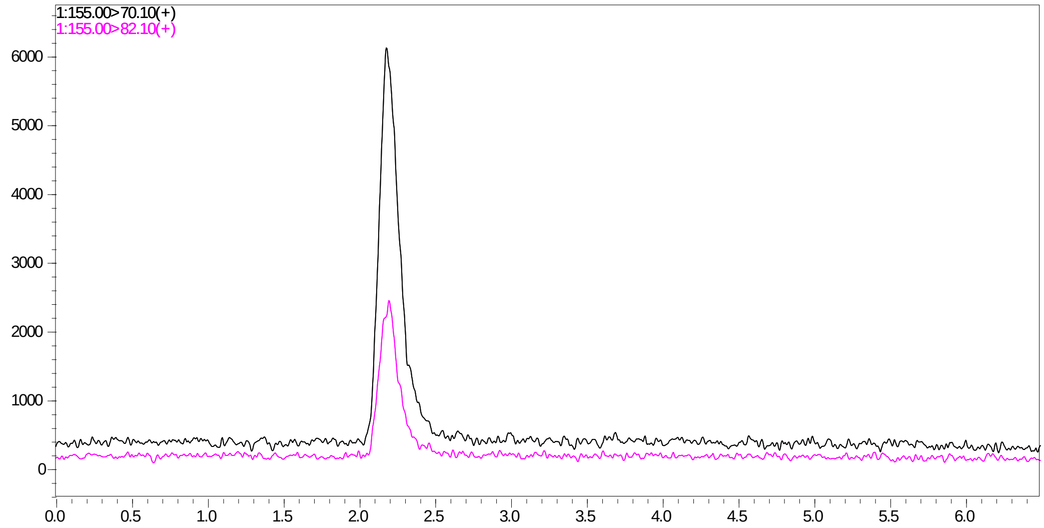

Figure S4. SRM chromatogram of the blank control group

Table S2. Screening of wild-type fungal strains from marine mangroves

Fungal species	Peak area	Peak area	Peak area	Relative abundance
Blank control	817461	825171	832169	-
P. incoloratum ma	3108263	2808440	3508229	2290802
P. pedernalense	11581374	104319174	109656461	10763913
P. steckii	7116047	7414681	6416450	6298585
P. MK351225.1	515711	505961	525472	-301750
P. citrinum	4695235	5095450	4595113	3877774
P. MK450735.1	387559	427672	337462	-429902
P. ehrlichii	1028872	1059139	1018674	211411
P. sumatraense	1563626	1623550	1513751	746165
A. versicolor	489368	499349	479461	-328093
A. alabamensis	2573915	2433114	2712700	1755454
A. sydowii	653339	653172	653340	-164122
A. reticulatus 1	748443	747364	748327	-69018
A. reticulatus 2	601909	601679	602040	-215552
A. gracilis	1119894	1120030	1119859	302433
A . terreus	746730	746473	746818	-70731

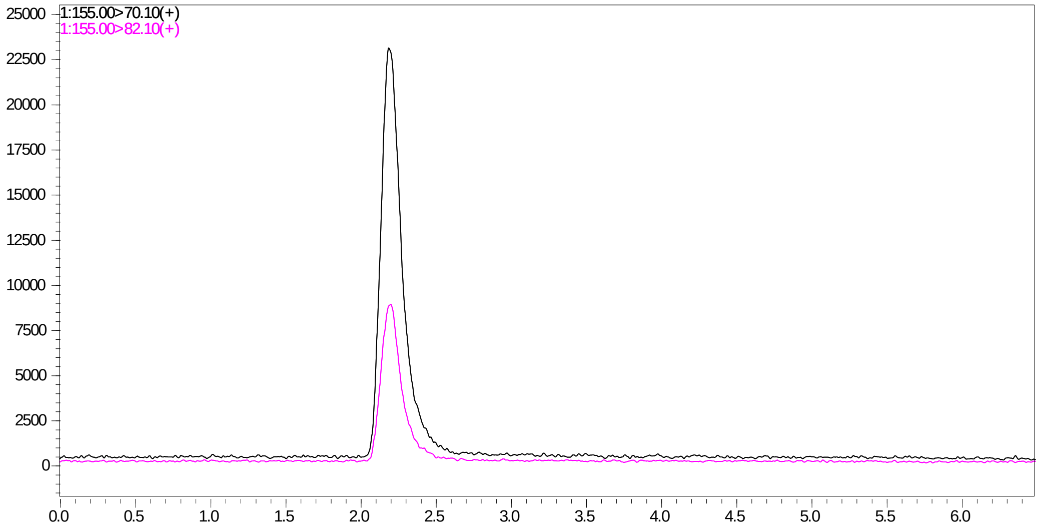

Figure S5. SRM chromatogram of the *Penicillium pedernalense*

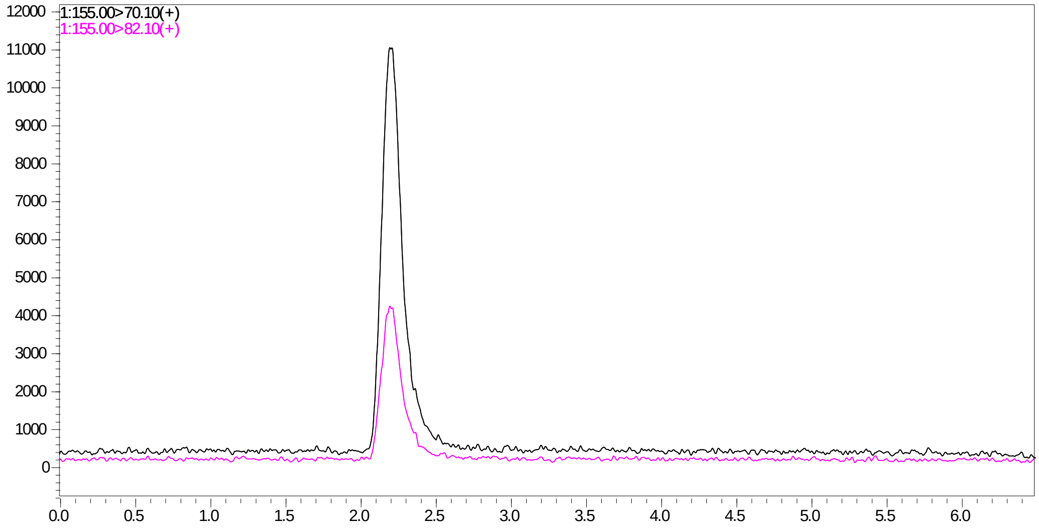

Figure S6. SRM chromatogram of the *Penicillium steckii*

Table S3. The limits of detection (LOD) and the limits of quantitation (LOQ)

	**Concentration of standard**	**Peak height**	**S/N**
	Blank solvent	480	-
**High concentration standard (ng/mL)**	100	29883	-
**LOD (ng/mL)**	4.8	1732	3.6
**LOQ (ng/mL)**	16.0	5366 (n=6)	11.2

Table S4. Calibration curve of the cGP standard

Concentration of standard	Peak area	Calibration curve	R^2
20	65163		
60	202016		
100	336259	y=3199x+9472	0.999
140	466212		
180	586826		
220	704240		

Table S5. The result of precision using a cGP standard solution at 20 μg/mL

	1	2	3	4	5	6	Average	SD	RSD%
**Peak Area(y)**	224667	223104	224146	224208	226049	232268	1.6	-	-
**Concentration/(x/ng • mL^{-1})**	67.269	66.781	67.107	67.126	67.701	69.646	67.605	1.043	1.54

Table S6 The result of repeatability using a cGP standard solution at 20 μg/mL

	1	2	3	4	5	6	Average	SD	RSD%
**Peak area**	222027	224571	221404	230639	228142	224667	1.6	-	-
**Concentration/(x/ng • mL^{-1})**	66.444	67.239	66.249	69.136	68.356	67.269	67.449	1.113	1.65

Table S7. The result of recovery

Sample concentration /ng • mL^{-1}	Spiked concentration /ng • mL^{-1}	Detection area	Detection concentration /ng • mL^{-1}	Recovery /%	Average recovery /%	RSD/ %
66.622	33.5	319059	96.776	90.01		
66.622	33.5	322159	97.745	92.91		
66.622	33.5	316884	96.096	87.98		
66.622	33.5	315737	95.738	86.91	88.62	2.9
66.622	33.5	314350	95.304	85.62		
66.622	33.5	317216	96.200	88.29		
66.622	67.0	412,405	125.956	88.56		
66.622	67.0	413,921	126.430	89.27		
66.622	67.0	415,383	126.887	89.94		
66.622	67.0	418,735	127.935	91.51	90.04	1.6
66.622	67.0	413,170	126.195	88.92		
66.622	67.0	419872	128.290	92.04		
